# Supplementary material for: From sequence to enzyme mechanism using multi-label machine learning
Source: BMC Bioinformatics. 2014 May 19;15:150. doi: 10.1186/1471-2105-15-150 (PMC4229970; doi:10.1186/1471-2105-15-150)
Supplement: Additional file 2 — Java code of ml2db. Additional file ml2db_code.tar.gz contains the Java source code to run the multi-label machine learning experiments and save the results to database. The code’s Javadoc is included. [file 1471-2105-15-150-S2.zip › additional file 2/ml2db/utils/doc/cern/jet/random/AbstractContinousDistribution.html]

AbstractContinousDistribution


JavaScript is disabled on your browser.


- Overview
- Package
- Class
- Use
- Tree
- Deprecated
- Index
- Help

- Prev Class
- Next Class

- Frames
- No Frames

- All Classes

- Summary:
- Nested |
- Field |
- Constr |
- Method

- Detail:
- Field |
- Constr |
- Method


cern.jet.random

## Class AbstractContinousDistribution

- java.lang.Object
- - cern.colt.PersistentObject
  - - cern.jet.random.AbstractDistribution
    - - cern.jet.random.AbstractContinousDistribution

- All Implemented Interfaces:
  :   DoubleFunction, IntFunction, java.io.Serializable, java.lang.Cloneable

  Direct Known Subclasses:
  :   Pareto

  ---

    

  ```
  public abstract class AbstractContinousDistribution
  extends AbstractDistribution
  ```

  Abstract base class for all continous distributions.

  Version:
  :   1.0, 09/24/99

  Author:
  :   wolfgang.hoschek@cern.ch

  See Also:
  :   Serialized Form

- - ### Method Summary

    - ### Methods inherited from class cern.jet.random.AbstractDistribution

      `apply, apply, clone, makeDefaultGenerator, nextDouble, nextInt`
    - ### Methods inherited from class java.lang.Object

      `equals, getClass, hashCode, notify, notifyAll, toString, wait, wait, wait`


- Overview
- Package
- Class
- Use
- Tree
- Deprecated
- Index
- Help

- Prev Class
- Next Class

- Frames
- No Frames

- All Classes

- Summary:
- Nested |
- Field |
- Constr |
- Method

- Detail:
- Field |
- Constr |
- Method
